# A nomogram incorporating functional and tubular damage biomarkers to predict the risk of acute kidney injury for septic patients

**DOI:** 10.1186/s12882-021-02388-w

**Published:** 2021-05-13

**Authors:** Jianchao Ma, Yujun Deng, Haiyan Lao, Xin Ouyang, Silin Liang, Yifan Wang, Fen Yao, Yiyu Deng, Chunbo Chen

**Affiliations:** 1 Department of Nephrology, Guangdong Provincial People’s Hospital, Guangdong Academy of Medical Sciences, 510080 Guangzhou, Guangdong PR China; 2 Department of Critical Care Medicine, Guangdong Provincial People’s Hospital, Guangdong Academy of Medical Sciences, 510080 Guangzhou, Guangdong Province PR China; 3 Department of Pharmacy, Guangdong Provincial People’s Hospital, Guangdong Academy of Medical Sciences, 106 Zhongshan Er Road, 510080 Guangzhou, Guangdong PR China; 4 Department of Intensive Care Unit of Cardiovascular Surgery, Guangdong Cardiovascular Institute, Guangdong Provincial People’s Hospital, Guangdong Academy of Medical Sciences, 106 Zhongshan Er Road, 510080 Guangzhou, PR China; 5 The Second School of Clinical Medicine, Southern Medical University, 510280 Guangzhou, Guangdong PR China

**Keywords:** Acute kidney injury, Sepsis, Serum cystatin C, Nomogram, N-acetyl-β-D-glucosaminidase, Intensive care unit

## Abstract

**Background:**

Combining tubular damage and functional biomarkers may improve prediction precision of acute kidney injury (AKI). Serum cystatin C (sCysC) represents functional damage of kidney, while urinary N-acetyl-β-D-glucosaminidase (uNAG) is considered as a tubular damage biomarker. So far, there is no nomogram containing this combination to predict AKI in septic cohort. We aimed to compare the performance of AKI prediction models with or without incorporating these two biomarkers and develop an effective nomogram for septic patients in intensive care unit (ICU).

**Methods:**

This was a prospective study conducted in the mixed medical-surgical ICU of a tertiary care hospital. Adults with sepsis were enrolled. The patients were divided into development and validation cohorts in chronological order of ICU admission. A logistic regression model for AKI prediction was first constructed in the development cohort. The contribution of the biomarkers (sCysC, uNAG) to this model for AKI prediction was assessed with the area under the receiver operator characteristic curve (AUC), continuous net reclassification index (cNRI), and incremental discrimination improvement (IDI). Then nomogram was established based on the model with the best performance. This nomogram was validated in the validation cohort in terms of discrimination and calibration. The decision curve analysis (DCA) was performed to evaluate the nomogram’s clinical utility.

**Results:**

Of 358 enrolled patients, 232 were in the development cohort (69 AKI), while 126 in the validation cohort (52 AKI). The first clinical model included the APACHE II score, serum creatinine, and vasopressor used at ICU admission. Adding sCysC and uNAG to this model improved the AUC to 0.831. Furthermore, incorporating them significantly improved risk reclassification over the predictive model alone, with cNRI (0.575) and IDI (0.085). A nomogram was then established based on the new model including sCysC and uNAG. Application of this nomogram in the validation cohort yielded fair discrimination with an AUC of 0.784 and good calibration. The DCA revealed good clinical utility of this nomogram.

**Conclusions:**

A nomogram that incorporates functional marker (sCysC) and tubular damage marker (uNAG), together with routine clinical factors may be a useful prognostic tool for individualized prediction of AKI in septic patients.

**Supplementary Information:**

The online version contains supplementary material available at 10.1186/s12882-021-02388-w.

## Key messages

The nomogram that incorporates functional marker (sCysC) and tubular damage marker (uNAG) effectively predicts AKI risk for septic patients in ICU.

## Background

Acute kidney injury (AKI) is frequent [1–3] and associated with poor prognosis [4–6]. Notably, one of the most common causes of AKI is sepsis [7] which is increasingly prevalent in critically ill patients [8–10]. Early recognition of AKI in septic patients may improve their clinical prognosis. Nevertheless, both AKI and sepsis are heterogeneous syndrome that represent multifactorial clinical conditions. In this context, early identification of AKI in septic patients remains a big challenge by using any single marker. Prior studies implied that combining markers of different characteristics may prove more accurate for AKI prediction in complex clinical settings [11–13].

Recently, combining functional and tubular damage biomarkers was reported to be an effective clinical strategy for AKI prediction, including patients suffering from heart failure [14] or those after cardiac surgery [15, 16]. Serum cystatin C (sCysC) is a glomerular filtration renal biomarker, while urinary N-acetyl-β-D-glucosaminidase (uNAG) represents tubular damage [12, 17]. NAG originates from the lysosomes of the renal proximal tubule cells and can be measured in the urine [12, 17]. Urinary NAG performed well as an early damage biomarker for AKI. In addition, previous study indicated that the increment of uNAG was caused not by sepsis, but by the occurrence of AKI [18].Moreover, both two biomarkers are clinically available [19]. Recently, we found that combining uNAG and sCysC was as an effective clinical strategy for detecting AKI in the postsurgical population [20]. Nevertheless, there is no nomogram containing this combination for predicting AKI in septic cohort.

Nomogram is used as a visualized, appreciable and intuitive tool for AKI prediction [21–23]. However, limited data exist on the nomogram for AKI prediction in septic patients. Furthermore, there are no prior nomograms incorporating uNAG or sCysC for AKI prediction in septic cohort. Therefore, this study aimed to compare the performance of AKI prediction models with or without incorporating the above-mentioned biomarkers and then develop an effective prediction nomogram for septic patients in intensive care unit (ICU).

## Materials and methods

### Study design and participants

This prospective observational study was conducted in the mixed medical-surgical ICU of a tertiary care hospital in China. All consecutive septic patients admitted to ICU from October 2014 to April 2019 were eligible for enrollment. The exclusion criteria included age under 18 years, presence of AKI before ICU admission, preexisting dialysis before ICU admission, end-stage renal disease (ESRD), renal transplantation, nephrectomy, refusal of consent, or missing data. The outcome was the detection of AKI within one week of ICU admission. The enrolled septic patients were divided into two cohorts in chronological order: development cohort and validation cohort. The protocol of this study was met Strengthening the Reporting of Observational Studies in Epidemiology [24] and Standards for Reporting Diagnostic Accuracy [25] criteria. 55oved by the local institutional review board.

### Sample and data collection

Similar to prior studies [19, 20], we simultaneously collected blood and urine samples within one hour after ICU enrollment. With the standard protocol, measurements of all samples were conducted at the central laboratory of the Guangdong Provincial People’s Hospital in 24 h after collection. The levels of uNAG and sCysC were measured once at the time of ICU admission. The detection of serum creatinine (sCr) was performed at ICU admission, and thenceforth at least once a day during ICU hospitalization. We prospectively collected baseline clinical characteristics of patients and recorded the hourly urine output from ICU admission to discharge.

The following clinical variables of patient were also recorded: sex, age, body mass index (BMI), preexisting clinical conditions of each patient, admission type, baseline sCr, baseline estimated glomerular filtration rate (eGFR),, blood laboratory values at ICU admission (hemoglobin, serum glucose, procalcitonin, lactate, C-reactive protein), Acute Physiology and Chronic Health Evaluation (APACHE) II score at ICU admission, Sequential organ failure assessment score (SOFA) at ICU admission, use of nephrotoxic drugs within 5 days before ICU admission (nonsteroidal anti-inflammatory drug, angiotensin-converting enzyme inhibitor, angiotensin receptor blocker, immunosuppressant, sulfadiazine, aminoglycoside, vancomycin, acyclovir, amphotericin, allopurinol, or polymyxin), administration of radiographic contrast before ICU admission, site of infection, mean arterial pressure at ICU admission, use of vasopressor at ICU admission (dopamine, norepinephrine, or vasopressin), length of ICU stay, length of hospital stay, renal replacement therapy (RRT) during ICU stay, 30-day mortality after ICU admission. The baseline estimated glomerular filtration rate (eGFR) was calculated by the Chronic Kidney Disease Epidemiology Collaboration Eq. [26].

### Definitions

The outcome was the development of AKI. According to the Kidney Disease Improving Global Outcomes (KDIGO) criteria for AKI [27], AKI was defined within one week after ICU admission as any of the following: increase in sCr by ≥ 0.3 mg/dl (≥ 26.5 µmol/l) within 48 h, or increase in sCr to ≥ 1.5 times baseline within one week, or urine output < 0.5 ml/kg/h for 6 h. Severe AKI was defined as KDIGO stage 2 or stage 3 AKI. A baseline creatinine was defined by the following rules ranked in the descending order of preference as previously recommended [28]: (1) the most recent pre-ICU creatinine value between 30 and 365 days before ICU admission (n = 58); (2) a stable pre-ICU creatinine value > 365 days for patients aged < 40 years, (the stable creatinine value was defined as within 15 % of the lowest ICU measurement) before ICU admission (n = 1); (3) pre-ICU creatinine value > 365 days before ICU admission and less than the initial sCr at ICU admission (n = 14); (4) a pre-ICU creatinine value (between 3 and 39 days before ICU admission) less than or equal to the initial on-admission creatinine to ICU and not distinctly in AKI (n = 153); (5) the lowest creatinine value upon initial admission to ICU (n = 54), the last ICU value (n = 51), or the minimum value at follow-up up to 365 days (n = 27). Sepsis was defined according to the American College of Chest Physicians and the Society of Critical Care Medicine Consensus Conference Committee guidelines [29].

### Biomarker assays

According to the manufacturer’s instructions, serum Cystatin C (CysC) and creatinine, urinary creatinine, and NAG levels were measured using the UniCel DxC 800 Synchron System (Beckman Coulter, USA). The coefficients of intraassay and interassay variation for sCysC were ≤ 10 % and < 5 %, respectively. The coefficients of intraassay and interassay variation of uNAG were both ≤ 10 %. The value of urinary NAG was normalized to urinary creatinine concentration. The personnel measuring the biomarkers were blinded to all patient’s clinical characteristics. The stability of sCysC and uNAG has already been proven [30–32], thereby pre-analysis about the influence of cooling or freezing of these samples was not performed.

### Sample size consideration

The sample size was calculated based on the rule described by Harrell, Vittinghoff, Steyerberg [33], namely, events per variable (EPV) being ten or greater was an important issue for estimation of multivariable regression coefficients in the multivariate regression model. Based on previous similar studies [19, 34], we considered about 5–6 significant clinical risk factors in developing a risk model of AKI. Therefore, it would need a minimum sample size of 50–60 (5*10 − 6*10) patients who had events (AKI) after ICU admission in development cohort.

### Statistical analysis

SPSS version 13.0 (SPSS, Chicago, IL, USA), R version 3.3.1 (R Foundation for Statistical Computing, Vienna, Austria), and MedCalc version 12.5.0 (MedCalc Software, Ostend, Belgium) software programs were used for statistical analysis.

Non-normally distributed continuous variables were presented as medians (interquartile range). The non-normally distributed continuous variables were compared by Wilcoxon rank-sum test or Kruskal-Wallis test for one-way analysis of variance. If the Kruskal–Wallis test showed statistical significance, a post hoc test was subsequently conducted with the Bonferroni correction. Categorical variables were expressed as number (percentage) and Chi-square or Fisher’s exact test was then used to compare the categorical variables. Non-parametric Spearman’s test was used to assess the correlations among continuous variables displayed non-normal distributions or categorical variables.

In order to assess the discrimination capability of clinical models for AKI prediction, receiver-operating characteristic (ROC) curve was generated. The area under the curve (AUC) was then derived from the ROC curve. All confidence interval (CI) presented are 95 %. The comparison of AUC between the groups in same data set was conducted with the method developed by DeLong et al. [35], and the comparison of AUC between the groups was conducted using Hanley-McNeil methods [36]. The optimal cut-off value for predicting AKI was defined according to the Youden’s index [37]. The specificity and sensitivity, negative and positive predictive values (NPV and PPV), and negative and positive likelihood ratios (LR) were also calculated.

To construct a predictive nomogram for the probability of AKI in septic patients, we firstly conducted univariate and multivariate logistic regression to construct the clinical models in the development cohort. The candidate variables with *P* < 0.10 in univariate analysis were included in multivariate analysis for further variable selection. The forward stepwise (likelihood ratio) was then used. Since the APACHE II score system not only overlaps with the SOFA score but also contains more evaluating parameters than the SOFA score. To avoid the collinearity and over-fitting, the APACHE II score but not the SOFA score was included in logistic regression. A clinical model was firstly constructed without candidate variables of uNAG and sCysC in univariate logistic regression. Then the performance of the two biomarkers (uNAG and sCysC) combined with this clinical model was compared by AUC, integrated discrimination improvement (IDI) index, and continuous net reclassification improvement (cNRI) index, as described previously [38, 39]. A predictive nomogram was obtained from the best one. The points of each factor in the nomogram were first gotten by drawing a vertical line from the predictor to the point axis. The total points for each patient were the sum of all the points from all the factors. The estimated probability of AKI occurrence was obtained by drawing a vertical line from the total point axis to the risk of AKI prediction. The Hosmer-Lemeshow test [40] was then used to evaluate the calibration plot of the nomogram. The validity of the predictive nomogram was verified in the validation cohort. Decision curve analysis (DCA) [41, 42] was also performed to evaluate the net benefit of decision for AKI prediction with the nomogram in the entire cohort.

All the tests were two-tailed, and *P* < 0.05 was considered statistically significant.

## Results

### Patient characteristics

Of the 713 consecutive adult septic patients screened for the inclusion in this study, 355 (49.8 %) were excluded (Additional file 1: Fig. S1). Thus, 358 patients were enrolled for analysis, including development cohort (232 patients) and validation cohort (126 patients). Patient characteristics are present in Table 1. In the development cohort, 69 patients (29.7 %) developed AKI after ICU admission, while 52 patients (41.3 %) developed AKI in the validation cohort. In entire cohort, there were 100 non-survivors within 30-day after ICU admission.

**Table 1:** Baseline characteristics and outcomes in entire cohort^a^

Characteristics	Development cohort (n = 232)	Validation cohort (n = 126)	*P*-value
**Demographic variables**			
Age, years	62 (50–72)	60 (51–72)	0.606
Males, n (%)	143 (61.6)	83 (65.9)	0.428
BMI, kg/m^2^	22.1 (20.3–23.9)	22.4 (19.6–25.7)	0.440
**Preexisting clinical conditions**			
Hypertension, n (%)	76 (32.8)	47 (37.3)	0.387
Diabetes mellitus, n (%)	34 (14.7)	18 (14.3)	0.925
Cerebrovascular disease, n (%)	63 (27.2)	46 (36.5)	0.066
Chronic liver disease, n (%)	11 (4.7)	2 (1.6)	0.220
Coronary artery disease, n (%)	23 (9.9)	17 (13.5)	0.305
Heart failure, n (%)	18 (7.8)	11 (8.7)	0.748
Malignancy, n (%)	51 (22.0)	29 (23.0)	0.823
CKD, n (%)	19 (8.2)	9 (7.1)	0.725
COPD, n (%)	28 (12.1)	13 (10.3)	0.619
**Admission type, n (%)**			0.112
Elective surgical, n (%)	38 (16.4)	14 (11.1)	
Emergency surgical, n (%)	44 (19.0)	17 (13.5)	
Medical, n (%)	150 (64.7)	95 (75.4)	
**Medication before ICU admission, n (%)**			
Nephrotoxic drugs^a^	45(19.4)	26(20.6)	0.779
Radiographic contrast	20(8.6)	15(11.9)	0.318
**Sites of infection, n (%)**			0.666
Pulmonary or thoracic cavity	163 (70.3)	3 (73.8)	
Abdomen	26 (11.2)	11 (8.7)	
Biliary tract	5 (2.2)	3 (2.4)	
CNS infections	19 (8.2)	6 (4.8)	
Others^b^	19 (8.2)	13 (10.3)	
MAP at ICU admission, mmHg	93 (84–104)	90 (82–101)	0.096
Need for vasopressor at ICU admission, n (%)	31(13.4)	25(19.8)	0.107
Mechanical ventilation at ICU admission, n (%)	143(61.6)	89(70.6)	0.089
**Laboratory test**			
Baseline serum creatinine, mg/dL	0.70 (0.57–0.87)	0.74 (0.59–0.89)	0.416
Baseline eGFR, mL/min/1.73 m^2^	96.01(81.98-111.62)	97.42(80.74–110.20)	0.997
sCysC at ICU admission, mg/L	0.93 (0.76–1.21)	0.99 (0.75–1.32)	0.271
uNAG at ICU admission, U/g Cre	44.21(24.07–74.25)	34.22 (20.60-61.52)	0.016
Serum creatinine at ICU admission, mg/dL	0.79 (0.66–1.01)	0.85 (0.69–1.03)	0.244
Serum glucose at ICU admission, mg/dL	143 (112–186)	150 (125–211)	0.060
Hemoglobin at ICU admission, g/L	110 (93–124)	106 (89–124)	0.420
Platelet at ICU admission,10^9^/L	197 (139–266)	188 (135–264)	0.903
Serum PCT at ICU admission,ng/ml	0.58 (0.16–2.89)	0.56 (0.18–2.55)	0.780
CRP at ICU admission,mg/L	58.66(18.33-124.48)	61.30(21.90-141.18)	0.638
Total bilirubin at ICU admission > 2 mg/dL, n (%)	30 (12.9)	19 (15.1)	0.572
Albumin at ICU admission < 3 mg/dL, n (%)	115(49.6)	47(37.3)	0.026
Lactate at ICU admission > 2mmol/L, n (%)	64 (27.6)	42 (33.3)	0.255
pH value at ICU admission ≤ 7.30, (%)	18 (7.8)	13 (10.3)	0.411
APACHE II score, at ICU admission	19 (15–24)	19 (15–24)	0.953
SOFA score, at ICU admission	5 (3–7)	4 (3–5)	< 0.001
UP^c^, ml/kg/h	1.77 (1.26–2.59)	1.66 (1.19–2.44)	0.128
**Outcomes**			
AKI, n (%)	69 (29.7)	52 (41.3)	0.028
**Severe AKI, n (%)**	**30 (12.9)**	**24 (19.0)**	**0.123**
RRT (during ICU stay), n (%)	12 (5.2)	19 (15.1)	0.001
ICU mortality, n (%)	47 (20.3)	18 (14.3)	0.161
In-hospital mortality, n (%)	53 (22.8)	20 (15.9)	0.118
30-day mortality, n (%)	64 (27.6)	36 (28.6)	0.843

^a^The non-normally distributed continuous variables are expressed as median (25th percentile to 75th percentile [interquartile range]). Categorical variables are expressed as n (%); ^b^includes any of the following medications administered within 5 days before ICU admission: nonsteroidal anti-inflammatory drug, angiotensin-converting enzyme inhibitor, angiotensin receptor blocker, immunosuppressant, sulfadiazine, aminoglycoside, vancomycin, acyclovir, amphotericin, allopurinol, or polymyxin; ^c^includes any of the following sites of infection: soft tissue, blood, or urinary tract; ^c^UP, urine production first 24 h after admission. Abbreviations:*AKI* acute kidney injury; *BMI* body mass index; *CKD* chronic kidney disease, defined as baseline eGFR<60 ml/min per 1.73m^2^; *COPD* chronic obstructive pulmonary disease; *CNS* central nervous system; *MAP* mean arterial pressure; *ICU* Intensive care unit; *eGFR* estimated glomerular filtration rate; *sCysC* serum Cystatin C; *uNAG* urinary N-acetyl-ß-D-glucosaminidase; *Cre* creatinine concentration; *PCT* procalcitonin; *CRP* C-reactive protein; *APACHE II* Acute Physiology and Chronic Health Evaluation score; *SOFA* sequential organ failure assessment score; *UP* urine production first 24 hours after admission; *RRT* renal replacement therapy.

### Development of the nomogram model for AKI prediction

The ROC curve analysis in development cohort revealed that both sCysC and uNAG predicted AKI with statistical significance (Additional file 2: Table S1). The AUC-ROC value of sCysC for AKI was 0.724, which was not superior to that of uNAG. The AUC-ROC value of the combination (sCysC and uNAG) for AKI was 0.781, which demonstrated better performance than either of these two individual biomarkers.

**Fig. 1:**
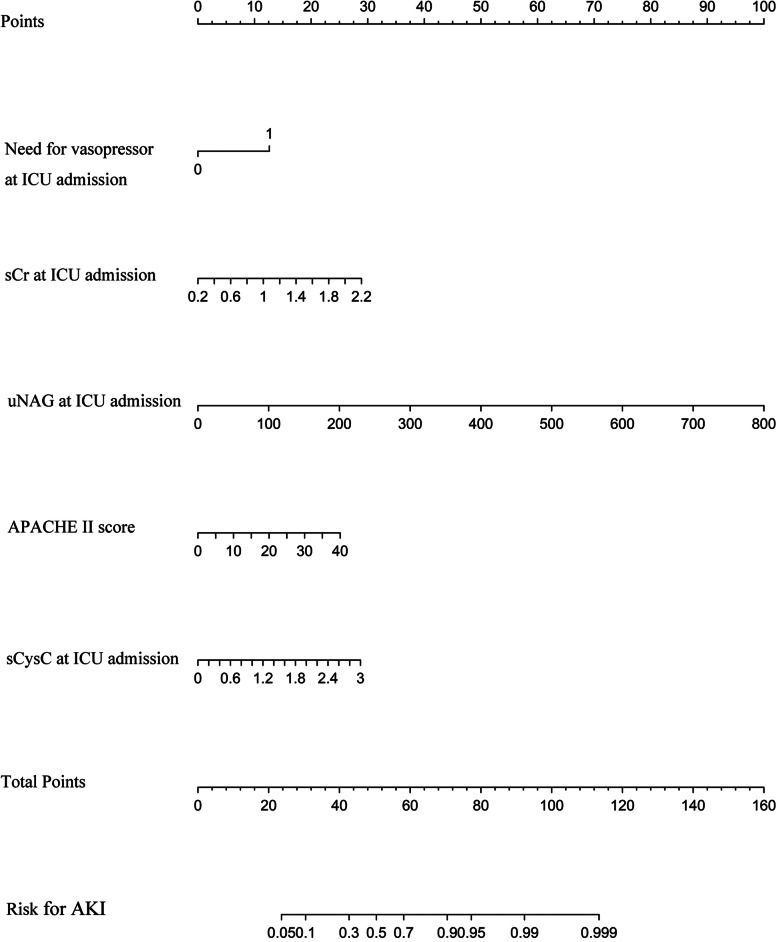
Nomogram predicting the probability of AKI in septic patients of the development cohort. Abbreviations: ICU, Intensive care unit; sCr, serum creatinine; sCysC, serum Cystatin C; uNAG, urinary N-acetyl-ß-D-glucosaminidase; APACHE II, Acute Physiology and Chronic Health Evaluation score

**Fig. 2:**
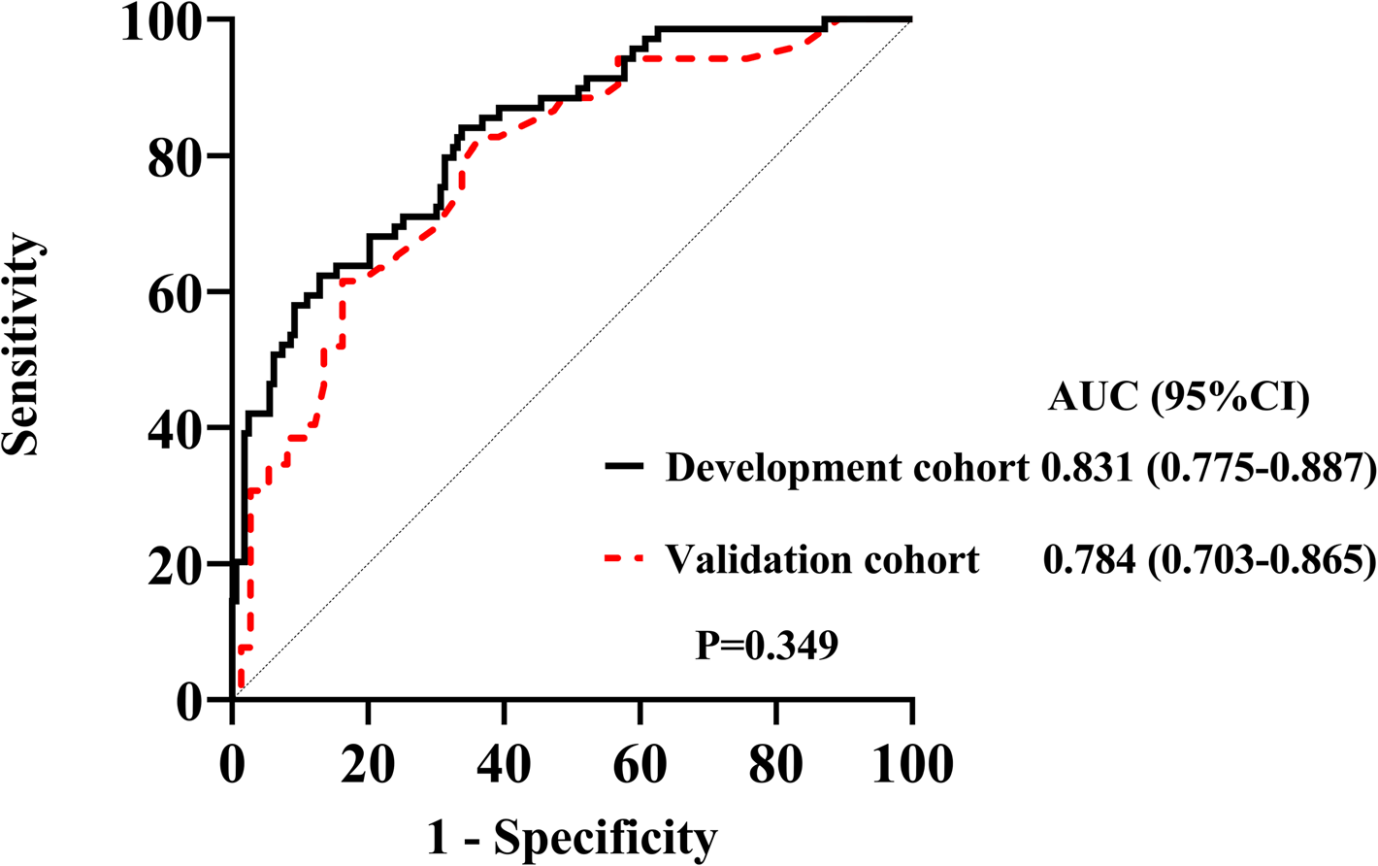
Receiver operating characteristic curve analyses of model for predicting the AKI in the development and validation cohort. Abbreviations: AKI, acute kidney injury; AUC, area under the receiver operator characteristic curve; CI, confidence interval

We first analyzed the risk factors for AKI prediction without candidate variables of uNAG and sCysC in univariate logistic regression (Table 2). The independent risk factors included APACHE II score, serum creatinine, and vasopressor used at ICU admission. The clinical model A for AKI prediction was then constructed. This model could predict AKI with reasonable certainty (AUC-ROC = 0.784). To evaluate the added contribution of these two biomarkers (uNAG and sCysC) to the clinical model for AKI prediction, logistic regression analysis was further performed (Table 3 and Additional file 3: Table S2). The AUC-ROC was significantly improved to 0.831 with the addition of uNAG plus sCysC (*P* = 0.034). The Hosmer-Lemeshow goodness-of-fit indicated that the risk model calibration was good (*P* = 0.383). Moreover, addition of sCysC plus uNAG significantly improved the risk reclassification of AKI over the clinical model A alone, with the largest cNRI (0.575) and IDI (0.085) (Table 3). Additionally, sCysC had significant but weak correlation with sCr at ICU admission (*P* < 0.01), while there was no significant correlation between uNAG and sCr at ICU admission (Additional file 4: Table S3). Therefore, the model containing sCysC, uNAG, serum creatinine, APACHE II score, and vasopressor used at ICU admission for AKI prediction was presented as the prediction nomogram (Fig. 1). ROC-AUC analyses for AKI prediction in the development cohort were showed in the Fig. 2.

**Table 2:** Logistic regression analysis of factors related to AKI in the development cohort^a^

Variable		Univariate analysis			Multivariate analysis	
	OR_unadj_	95 % CI	*P* value	OR_adj_	95 % CI	*P* value
Age, years	1.006	0.989–1.025	0.476			
Males	0.622	0.351–1.102	0.104			
BMI, kg/m2	0.985	0.901–1.077	0.735			
Preexisting clinical conditions						
Hypertension	1.248	0.690–2.256	0.464			
Diabetes mellitus	1.349	0.626–2.907	0.444			
Cerebrovascular disease	1.696	0.919–3.128	0.091			
Chronic liver disease	2.044	0.602–6.937	0.251			
Coronary artery disease	1.956	0.813–4.704	0.134			
Heart failure	1.560	0.578–4.210	0.380			
Malignancy	0.669	0.326–1.374	0.274			
CKD	3.675	1.408–9.591	0.008			
COPD	1.137	0.487–2.655	0.767			
Admission type, n (%)			0.064			
Elective surgical (reference)						
Emergency surgical	3.080	0.990–9.578	0.052			
Medical	3.300	1.214–8.970	0.019			
Medication before ICU admission, n (%)						
Nephrotoxic drugs^b^	1.084	0.535–2.194	0.823			
Radiographic contrast	2.073	0.818–5.255	0.125			
Sites of infection, n (%)			0.982			
Pulmonary or thoracic cavity (reference)						
Abdomen	1.065	0.434–2.615	0.891			
Biliary tract	1.597	0.259–9.864	0.614			
CNS infections	0.856	0.292–2.508	0.776			
Others^c^	1.106	0.397–3.080	0.847			
MAP at ICU admission, mmHg	1.002	0.985–1.019	0.833			
Need for vasopressor at ICU admission	6.694	2.948–15.196	< 0.001	5.637	2.349–13.528	< 0.001
Mechanical ventilation at ICU admission	1.925	0.730–5.071	0.185			
Serum creatinine at ICU admission, mg/dL	7.732	2.784–21.472	< 0.001	8.955	2.775–28.903	< 0.001
Serum glucose at ICU admission, mg/dL	1.004	0.999–1.008	0.093			
Hemoglobin at ICU admission, g/L	0.996	0.985–1.007	0.454			
Platelet at ICU admission,10^9^/L	0.997	0.994-1.000	0.070			
Serum PCT at ICU admission,ng/ml	1.014	0.994–1.034	0.170			
CRP at ICU admission,mg/L	1.002	0.998–1.006	0.287			
Total bilirubin at ICU admission > 2 mg/dL	1.994	0.909–4.371	0.085			
Albumin at ICU admission < 3 mg/dL	1.160	0.660–2.038	0.606			
Variable		**Univariate analysis**			**Multivariate analysis**	
	**OR**_**unadj**_	**95 % CI**	***P*value**	**OR**_**adj**_	**95 % CI**	***P*value**
Lactate at ICU admission > 2mmol/L	0.996	0.531–1.871	0.991			
pH value at ICU admission ≤ 7.30	1.198	0.431–3.334	0.729			
APACHE II score	1.106	1.057–1.156	< 0.001	1.104	1.050–1.160	< 0.001
UP, ml/kg/h	1.142	0.893–1.460	0.289			

^a^The clinical model was constructed without candidate variables of uNAG and sCysC in univariate logistic regression

^b^includes any of the following medications administered within 5 days before ICU admission: nonsteroidal anti-inflammatory drug, angiotensin-converting enzyme inhibitor, angiotensin receptor blocker, immunosuppressant, sulfadiazine, aminoglycoside, vancomycin, acyclovir, amphotericin, allopurinol, or polymyxin; ^c^includes any of the following sites of infection: soft tissue, blood, or urinary tract. Abbreviations: *AKI* acute kidney injury; *OR*_*unadj*_ odds ratio unadjusted; *OR*_*adj*_ odds ratio adjusted; *CI* confidence interval; *BMI* body mass index; *CKD* chronic kidney disease, defined as baseline eGFR<60 ml/min per 1.73m^2^; *COPD* chronic obstructive pulmonary disease; *CNS* central nervous system; *MAP* mean arterial pressure; *ICU* Intensive care unit; *eGFR* estimated glomerular filtration rate; *sCysC* serum Cystatin C; *uNAG* urinary N-acetyl-ß-D-glucosaminidase; *Cre* creatinine concentration; *PCT* procalcitonin; *CRP* C-reactive protein; *APACHE II* Acute Physiology and Chronic Health Evaluation score; *UP* urine production first 24 hours after admission; *RRT* renal replacement therapy.

**Table 3:** AUC-ROC, NRI and IDI analyses of AKI in development cohort

Variables	AUC-ROC	*P-*value^b^	IDI (95 % CI)	*P-*value^b^	cNRI (95 % CI)	*P-*value^b^
Clinical model A^**a**^	0.784(0.720–0.849)					
+uNAG	0.817(0.759–0.876)	0.091	0.068(0.028–0.108)	< 0.001	0.504(0.231–0.776)	< 0.001
+sCysC	0.807(0.747–0.867)	0.142	0.034(0.004–0.063)	0.024	0.468(0.193–0.743)	< 0.001
+sCysC and uNAG	0.831(0.775–0.887)	0.034	0.085(0.042–0.128)	< 0.001	0.575(0.303–0.847)	< 0.001

^**a**^Clinical model A for AKI prediction is composed of serum creatinine at ICU admission, need for vasopressor at ICU admission, APACHE II score; ^**b**^Versus clinical model A. Abbreviations: *AKI* acute kidney injury; *AUC-ROC* area under the receiver operating characteristic curve; *NRI* net reclassification improvement index; *IDI* integrated discrimination improvement index; *CI* Confidence Interval; *sCysC* serum Cystatin C; *uNAG* urinary N-acetyl-ß-D-glucosaminidase; *ICU* intensive care unit; *APACHE II* Acute Physiology and Chronic Health Evaluation score.

Severe AKI can also be distinguished by the prediction model which included sCysC and uNAG. The AUC for severe AKI is 0.741 (0.675–0.808) in the entire cohort. However, there was no significant difference in nomogram score between mild and severe AKI patients (Additional file 5: Table S4).

### Validation of the nomogram model for AKI prediction

**Fig. 3:**
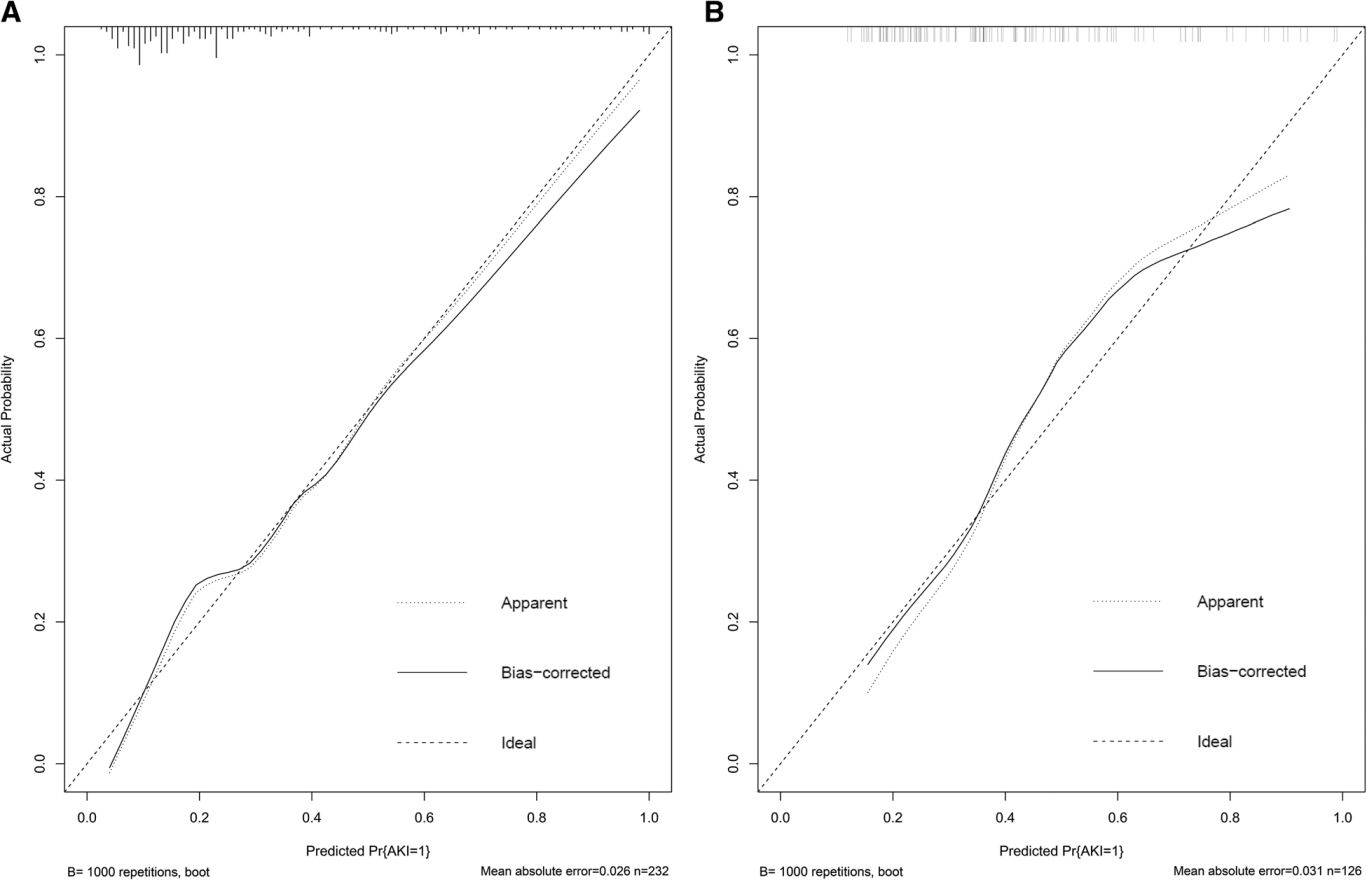
Calibration plot for nomogram in the development (A) and validation cohort(B). In the calibration plot, the X-axis represents the predicted probability of AKI, and the Y-axis indicates the actual AKI rate. The 45º dashed line illustrates ideal predictions, the plot represents the accuracy of the best-fit model (“Apparent”) and the bootstrap model (“Bias-corrected”) for predicting AKI. The calibration plot illustrates the relationship between the predicted probability and observed probability of the scoring system for predicting AKI in the data set. Abbreviations: AKI, acute kidney injury

Based on the clinical models constructed in the development cohort, we compared the predictive ability of these two models in the validation cohort (Additional file 6: Table S5). In validation cohort, the clinical model including the APACHE II score, serum creatinine, and vasopressor used at ICU admission yielded AUC of 0.668 (95 % CI 0.570–0.765). Adding sCysC and uNAG to this model significantly improved the AUC to 0.784 (95 %CI 0,703-0.865) (*P* < 0.001). Furthermore, incorporating them significantly improved risk reclassification over the predictive model alone, with cNRI (0.660) and IDI (0.104). Therefore, the model containing sCysC, uNAG, serum creatinine, APACHE II score, and vasopressor used at ICU admission was presented as the prediction nomogram for AKI (Fig. 1). Correspondingly, ROC analysis of this model for AKI in the validation cohort was demonstrated (Fig. 2). The *P* value for the Hosmer-Lemeshow goodness-of-fit of this model in the validation cohort were 0.541. The AUC-ROC (95 %CI) for the development and validation cohort was 0.831 (0.775–0.887) and 0.784(0.703–0.865), respectively. There was no significant difference between them (*P* = 0.349). First, the clinical model was internally validated with the bootstrap validation method (Fig. 3 a). Calibration plots for the nomogram in the development (Fig. 3 a) and validation (Fig. 3b) cohort were then generated. The calibration plots demonstrated that the AKI predicted probabilities of AKI agreed with the actual probabilities.

**Fig. 4:**
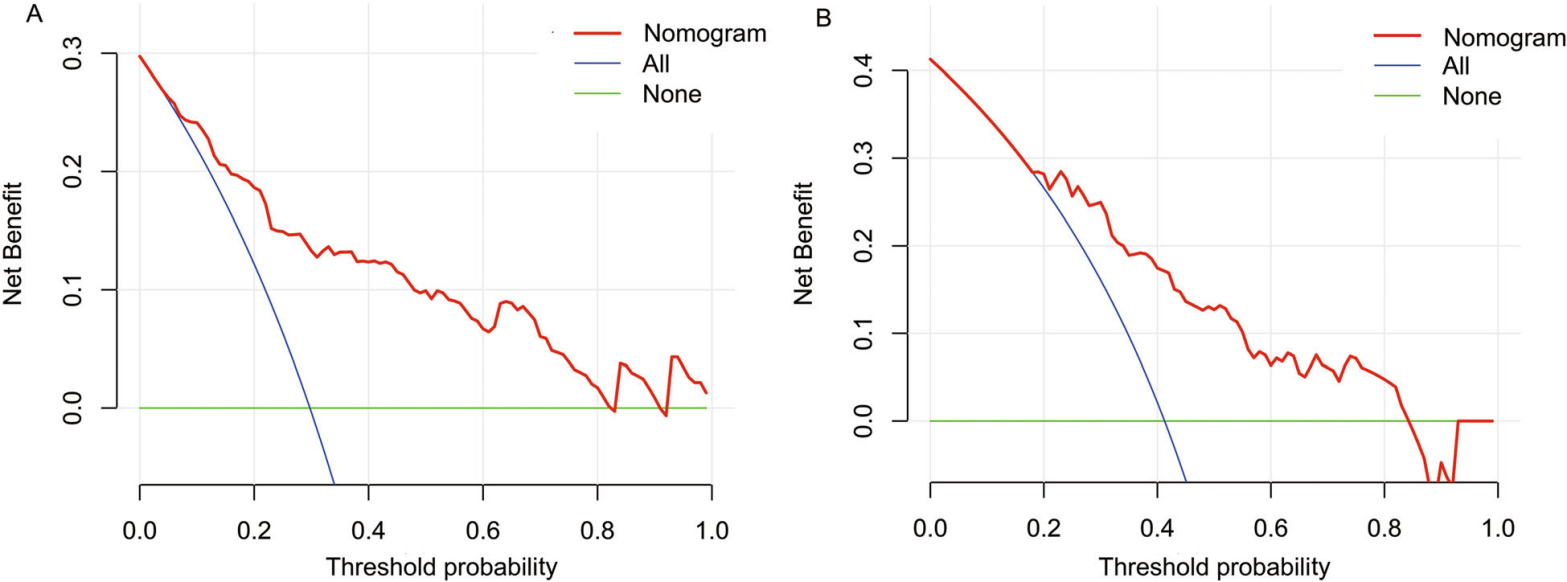
DCA of the nomogram for AKI prediction in both development and validation cohorts. (A) The DCA of nomogram in development cohort; (B) The DCA of nomogram in validation cohort. The Y-axis shows the net benefit, and the X-axis indicates the threshold probability. The red line represents the nomogram. The blue line indicates the assumption that all patients are suffered from AKI and undertaken treatment. The green line represents the assumption that no patient is suffered from AKI and undertaken treatment

The DCA demonstrated that nomogram could add more net benefits over the “treat-none” or “treat-all” strategies, which indicated good clinical utility of this nomogram (Fig. 4).

### Nomogram and adverse outcome

Notably, there was significantly positive relationship between mortality and the total score calculated by the AKI risk nomogram (Additional file 7: Table S6). Moreover, there was significantly negative relationship between the nomogram score and the timing of AKI occurrence (r = -0.264, *P* = 0.003) (Additional file 8: Table S7).

SOFA predicted AKI in development cohort with a sensitivity of 52 % and a specificity of 79 %, respectively (Additional file 9: Table S8). Given the correlation between SOFA score and sepsis, we further constructed a prediction model in the development cohort with SOFA score as one of the candidate variables instead of APACHE II (Additional file 10: Table S9). A prediction model for AKI prediction was then built including serum creatinine at ICU admission, need for vasopressor at ICU admission, SOFA score, sCysC, and uNAG (Additional file 11: Table S10). The model yielded an AUC of 0.830 in the development cohort, and 0.776 in the validation cohort (Additional file 12: Table S11). There was no significant difference between them.

## Discussion

The main finding of the present study was that the prediction nomogram incorporating renal functional marker (sCysC) and tubular damage marker (uNAG), together with routine clinical factors may be a useful tool for individualized prediction of AKI in septic patients. To the best of our knowledge, this study demonstrates for the first time that the nomogram incorporating sCysC and uNAG yields good discrimination for AKI prediction in septic patients.

AKI, one of the most frequent complication of critically ill patients, is especially common in septic patients [7]. In our septic cohort, AKI prevalence after ICU admission was 33.8 %. Hitherto, no single marker can reflect the complexity of the pathogenesis of AKI [43]. Accordingly, the ADQI working group recommended biomarkers combination for improvement in the recognition of AKI [13], including combining tubular damage and functional biomarkers. Urinary NAG represents tubular damage [12, 17], while sCysC is considered as functional biomarker [17]. Notably, they are clinically available. Moreover, sCysC was reported to be associated with AKI development in septic patients [44]. Additionally, previous study indicated that the increment of uNAG was caused not by sepsis, but by the occurrence of AKI [18]. Hence, we selected them to conduct our investigation based on the hypothesis that renal biomarkers can be classified as those indicating tubular damage and those representing changes in renal function [17]. In the present study, we found that compared to the clinical model without sCysC and uNAG, the model incorporating them performed better for AKI prediction in septic patients. The improved ability of these two biomarkers (sCysC and uNAG) in this study is in keeping with our prior study in patients undergoing neurosurgery [20].

Therefore, we developed and validated a nomogram for AKI risk prediction including above-mentioned biomarkers. Previous studies reported several nomogram models in predicting AKI [21, 23], however, seldom focused on septic patients. Recently, an analysis identified predictive factors in septic patients admitted to the ICU in the first 24-hour and constructed a nomogram for AKI [45]. In that study, only traditional clinical parameters were considered without new renal biomarkers. To our knowledge, our nomogram is the first one incorporating functional and tubular damage biomarkers for AKI prediction in septic patients. Our nomogram effectively predicted AKI risk as indicted by the AUC-ROC value. The bootstrapped calibration curves also demonstrated that the prediction agreed well with the actual observation of AKI.

The foremost usage of this nomogram is to individually predict the probability of AKI occurrence in septic patients. The points of each risk factor in this nomogram were first determined by drawing a vertical line from the predictor to the point axis. Second, all the points from all the risk factors were sum up to generate the total points. Third, the estimated probability of AKI could be obtained by drawing a vertical line from the total point axis to the risk of AKI. For example, a patient who need for vasopressor at ICU admission (corresponds to 12 points) has serum creatinine at ICU admission of 0.6 mg/dL (5 points), uNAG at ICU admission of 98 U/g Cr (10 points), sCySc at ICU admission of 1.2 mg/L (10 points), and APACHE II score of 26 (15 points). According to the proposed nomogram, the final point is calculated as the sum of scores for all risk factors (12 + 5 + 10 + 10 + 15 = 52), predicting AKI risk of approximately 60 %. To validate the clinical utility of this nomogram, we employed DCA to assess the nomogram in the entire cohort. Based on threshold probability, this novel statistic method provided further insight into clinical consequences and calculated the net benefit gained from the nomogram. In the present study, the DCA indicated that the proposed nomogram had good clinical utility. Therefore, this nomogram could facilitate doctors an advisable decision before any administration of prevention or treatment.

CKD is associated with AKI occurrence [46]. However, CKD was not retained in the present risk model. The APACHE II score, including but not limited to patient’s serum creatinine and chronic kidney function status, is a physiologically based system containing 12 physiological parameters. Therefore, APACHE II score is a common prediction tool of adverse outcome in ICU patients. In the present study, APACHE II was chosen as one of the independent predictors in the risk model. Probably owing to this reason, CKD was not chosen during the multivariate logistic regression in our study.

Our study has limitations. First, we only measured these two biomarkers once at ICU admission. According to ADQI recommendation [13], it may be not practical and cost-effective for collecting and measuring a series of samples at frequent time points. Therefore, we speculate that our conclusions are not debilitated by this limitation. Second, without an external validation dataset, we could not assess whether our nomogram may be suitable to patients outside of our center. Future study including multicenter is need. Third, there was significant but weak correlation between sCysC and sCr at ICU admission. Hence, multicollinearity of the risk factors still should be concerned in our study, even though we used multivariate logistic regression for further variable selection. Last but not least, nephrotoxins exposure was not included in our risk model. We constructed an AKI nomogram with readily available variables obtained at ICU admission for clinicians to screen the high-risk patients. Accordingly, nephrotoxins exposure after ICU admission was not taken into account in this study, which may partly contribute to the exclusion of nephrotoxins exposure. The study regarding the renal effects of nephrotoxins exposure will be conducted in future study.

The performance of AKI risk model may differ considerably across different clinical settings. Our future studies will focus on the comparison between other published models and our proposed nomogram. In addition, more recent statistical techniques, such as logistic least absolute shrinkage and selection operator (LASSO) regression need to be applied in future study.

## Conclusions

The present study showed that a prediction nomogram that incorporates functional marker (sCysC) and tubular damage marker (uNAG), together with routine clinical factors may be an effective tool for individualized prediction of AKI in septic patients.

## Supplementary Information

**Additional file 1: Supplementary Figure 1.**Flow chart from recruitment to outcome. Abbreviations: ICU, intensive care unit; AKI, acute kidney injury.

Additional file 2:  **(Table S1.)** Predictive characteristics of two biomarkers and their combination for AKI prediction in the development cohort.

Additional file 3: **(Table S2.) **Multivariate Logistic regression analysis of factors related to AKI in the development cohort.

Additional file 4: **(Table S3.) **Correlations among the risk factors in the prediction model for AKI in the development cohort.

**Additional file 5: Table S4.** Nomogram for predicting severe AKI.

Additional file 6: **(Table S5.) **AUC-ROC, NRI and IDI analyses of AKI in the validation cohort.

Additional file 7: **(Table S6.) **Correlations between AKI and mortality in entire cohort.

Additional file 8: **(Table S7.) **Correlation between the total score calculated from the nomogram and thedayof AKIoccurrence after ICU admission.

Additional file 9 **(Table S8.) **Predictive characteristics of SOFA and APCHE II for AKI prediction in the development cohort.

Additional file 10: **(Table S9.) **Logistic regression analysis of factors related to AKI in the development cohort including SOFA.

Additional file 11: **(Table S10.) **AUC-ROC, NRI and IDI analyses of AKI in development cohort.

Additional file 12: **(Table S11.) **AUC-ROC of the AKI prediction model in the development cohort and the validation cohort.

## Data Availability

The cohorts generated and/or analyzed during this study are not publicly available, due to currently ongoing research studies, but the data are available from the corresponding author on reasonable request.

## Abbreviations

AKI: Acute kidney injury
sCysC: Serum cystatin C
uNAG: Urinary N-acetyl-β-D-glucosaminidase
sCr: Serum creatinine
ICU: Intensive care unit
ESRD: End-stage renal disease
BMI: Body mass index
eGFR: Estimated glomerular filtration rate
APACHE II: Acute Physiology and Chronic Health Evaluation score
RRT: Renal replacement therapy
MDRD: Modification of Diet in Renal Disease formula
KDIGO: Kidney Disease Improving Global Outcomes criteria
AUC-ROC: Area under the receiver operating characteristic curve
CI: Confidence Interval
PPV: Positive predictive value
NPV: Negative predictive value
(+) LR: Positive likelihood ratio
(-) LR: Negative likelihood ratio
NRI: Net reclassification improvement index
IDI: Integrated discrimination improvement index
DCA: Decision curve analysis
DM: Diabetes mellitus
CAD: Coronary artery disease
COPD: Chronic obstructive pulmonary disease
HF: Heart failure
CKD: Chronic kidney disease, defined as baseline eGFR<60 ml/min per 1.73m^2^
PCT: Procalcitonin
CRP: C-reactive protein
SOFA: Sequential organ failure assessment score
UP: Urine production first 24 hours after admission
