# Supplementary material for: A nomogram incorporating functional and tubular damage biomarkers to predict the risk of acute kidney injury for septic patients
Source: BMC Nephrol. 2021 May 13;22:176. doi: 10.1186/s12882-021-02388-w (PMC8120900; doi:10.1186/s12882-021-02388-w)
Supplement: Supplementary file 1 — Additional file 1: Supplementary Figure 1.Flow chart from recruitment to outcome. Abbreviations: ICU, intensive care unit; AKI, acute kidney injury. [file 12882_2021_2388_MOESM1_ESM.pdf]

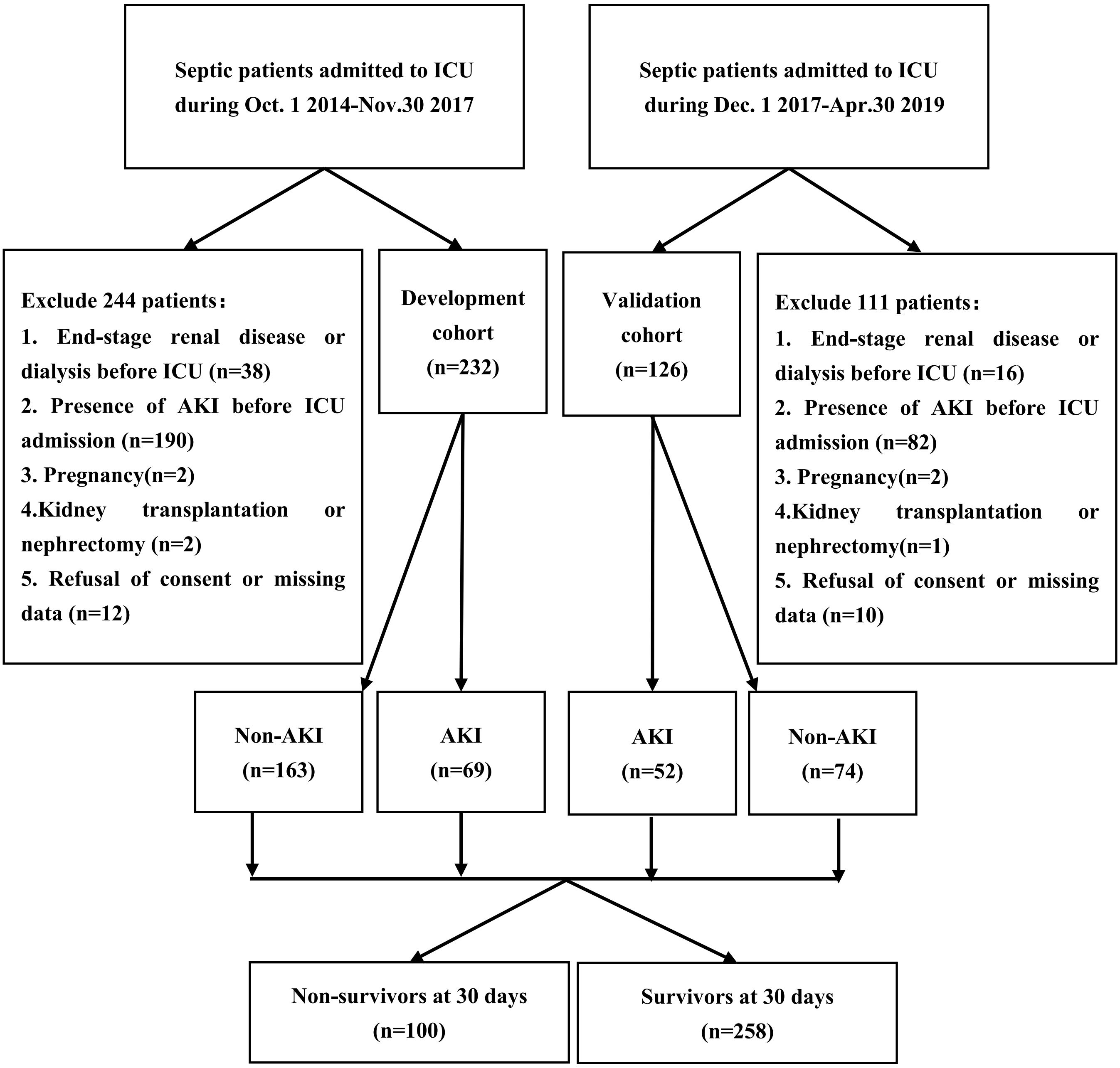

Supplementary Figure 1. Flow chart from recruitment to outcome.

Abbreviations: ICU, intensive care unit; AKI, acute kidney injury.
