# Supplementary material for: A nomogram incorporating functional and tubular damage biomarkers to predict the risk of acute kidney injury for septic patients
Source: BMC Nephrol. 2021 May 13;22:176. doi: 10.1186/s12882-021-02388-w (PMC8120900; doi:10.1186/s12882-021-02388-w)
Supplement: Supplementary file 2 — (Table S1.) Predictive characteristics of two biomarkers and their combination for AKI prediction in the development cohort. [file 12882_2021_2388_MOESM2_ESM.docx]

**Supplementary Table 1 Predictive characteristics of two biomarkers and their combination for AKI prediction in the development cohort**

| **Logistic regression model** | **AUC-ROC^a^** | **Cut-off^b^** | **Se** | **Sp** | **+LR** | **-LR** | **PPV** | **NPV** |
| --- | --- | --- | --- | --- | --- | --- | --- | --- |
| **AKI (n = 69)** |  |  |  |  |  |  |  |  |
| **Univariate models** |  |  |  |  |  |  |  |  |
| **sCysC** | 0.724(0.652-0.797)^#^ | 1.00mg/L | 0.70 | 0.68 | 2.18 | 0.45 | 0.48 | 0.84 |
| **uNAG** | 0.726(0.657-0.795)^#^ | 31.83U/g Cre | 0.88 | 0.44 | 1.58 | 0.26 | 0.40 | 0.90 |
| **Multivariate models** |  |  |  |  |  |  |  |  |
| **sCysC+ uNAG** | 0.781(0.717-0.846) | 0.39^c^ | 0.55 | 0.91 | 5.98 | 0.49 | 0.72 | 0.83 |

**^a^**Values are presented as AUC-ROC (95% confidence interval); **^b^**Ideal cut-off value according to Youden’s index; ^c^Cut-off points of the biomarker panel was the predicted probability generated from the multiple logistic regression model; ^#^*P*<0.05 vs. sCysC + uNAG.

**Abbreviations:** AKI, Acute kidney injury; AUC-ROC, area under the receiver operating characteristic curve; (+) LR, positive likelihood ratio; (-) LR, negative likelihood ratio; PPV, positive predictive value; NPV, negative predictive value; sCysC, serum Cystatin C; uNAG, urinary N-acetyl-ß-D-glucosaminidase; Cre, creatinine concentration.
