# Supplementary material for: A nomogram incorporating functional and tubular damage biomarkers to predict the risk of acute kidney injury for septic patients
Source: BMC Nephrol. 2021 May 13;22:176. doi: 10.1186/s12882-021-02388-w (PMC8120900; doi:10.1186/s12882-021-02388-w)
Supplement: Supplementary file 3 — (Table S2.) Multivariate Logistic regression analysis of factors related to AKI in the development cohort. [file 12882_2021_2388_MOESM3_ESM.docx]

**Supplementary Table 2 Multivariate Logistic regression analysis of factors related to AKI in the development cohort***

| Variable | Regression coefficient |  | OR_adj_ | 95% CI | *P* value |
| --- | --- | --- | --- | --- | --- |
| Need for vasopressor at ICU admission | 1.384 |  | 3.989 | 1.548-10.278 | 0.004 |
| sCysC at ICU admission, mg/L | 1.051 |  | 2.860 | 1.126-7.264 | 0.027 |
| uNAG at ICU admission, U/g Cre | 0.014 |  | 1.014 | 1.006-1.022 | 0.001 |
| Serum creatinine at ICU admission, mg/dL | 1.585 |  | 4.881 | 1.229-19.382 | 0.024 |
| APACHE II score | 0.069 |  | 1.071 | 1.016-1.130 | 0.011 |
| Intercept | -5.854 |  | 0.003 |  | <0.001 |

**Abbreviations:** AKI, acute kidney injury; OR_unadj_, odds ratio unadjusted; OR_adj_, odds ratio adjusted; CI, confidence interval; ICU, Intensive care unit; sCysC, serum Cystatin C; uNAG, urinary N-acetyl-ß-D-glucosaminidase; Cre, creatinine concentration; APACHE II, Acute Physiology and Chronic Health Evaluation score.
