# Supplementary material for: A nomogram incorporating functional and tubular damage biomarkers to predict the risk of acute kidney injury for septic patients
Source: BMC Nephrol. 2021 May 13;22:176. doi: 10.1186/s12882-021-02388-w (PMC8120900; doi:10.1186/s12882-021-02388-w)
Supplement: Supplementary file 4 — (Table S3.) Correlations among the risk factors in the prediction model for AKI in the development cohort. [file 12882_2021_2388_MOESM4_ESM.docx]

**Supplementary Table 3 Correlations among the risk factors in the prediction model for AKI in the development cohort**

| **Spearman’s rho** | **sCr(mg/dL)** | **sCysC (mg/L)** | **uNAG (U/g Cre)** |
| --- | --- | --- | --- |
| sCr(mg/dL) | 1 |  |  |
| sCysC (mg/L) | 0.395^a^ | 1 |  |
| uNAG (U/g Cre) | -0.124^b^ | 0.249^a^ | 1 |

Correlations **^a^***P* <0.01, **^b^***P* >0.05.

**Abbreviations:** AKI, acute kidney injury; sCr, serum creatinine at ICU admission; sCysC, serum Cystatin C; uNAG, urinary N-acetyl-ß-D-glucosaminidase; Cre, creatinine concentration.
