# Supplementary material for: A nomogram incorporating functional and tubular damage biomarkers to predict the risk of acute kidney injury for septic patients
Source: BMC Nephrol. 2021 May 13;22:176. doi: 10.1186/s12882-021-02388-w (PMC8120900; doi:10.1186/s12882-021-02388-w)
Supplement: Supplementary file 5 — Additional file 5: Table S4. Nomogram for predicting severe AKI. [file 12882_2021_2388_MOESM5_ESM.docx]

**Supplementary Table 4 Nomogram^a^ for predicting severe AKI**

| **Variables** | **Non-AKI**  **(n=237)** | **Mild AKI**  **(n=67)** | **Severe AKI**  **(n=54)** | **AUC-ROC^b^**  **(95% CI)** |
| --- | --- | --- | --- | --- |
| Nomogram score | 34 (29-40) | 50 (38-63)**^c^** | 48 (39-59)**^c^** | 0.741 (0.675-0.808) |

**^*^**The non-normally distributed continuous variables are expressed as median (25th percentile to 75th percentile [interquartile range]); ^a^The nomogram was calculated form the proposed clinical model for AKI prediction. This model is composed of serum creatinine at ICU admission, need for vasopressor at ICU admission, APACHE II score, sCysC, and uNAG; **^b^**Values are presented as AUC-ROC (95% confidence interval).

**^c^***P*<0.05 vs. Non-AKI.

**Abbreviations:** AKI, acute kidney injury; AUC-ROC, area under the receiver operating characteristic curve; CI, Confidence Interval; sCysC, serum Cystatin C; uNAG, urinary N-acetyl-ß-D-glucosaminidase; ICU, intensive care unit; APACHE II, Acute Physiology and Chronic Health Evaluation score.
