# Supplementary material for: A nomogram incorporating functional and tubular damage biomarkers to predict the risk of acute kidney injury for septic patients
Source: BMC Nephrol. 2021 May 13;22:176. doi: 10.1186/s12882-021-02388-w (PMC8120900; doi:10.1186/s12882-021-02388-w)
Supplement: Supplementary file 6 — (Table S5.) AUC-ROC, NRI and IDI analyses of AKI in the validation cohort. [file 12882_2021_2388_MOESM6_ESM.docx]

**Supplementary Table 5 AUC-ROC, NRI and IDI analyses of AKI in the validation cohort**

| **Variables** | **AUC-ROC** | ***P-*value^a^** | **IDI (95% CI)** | ***P-*value^a^** | **cNRI (95% CI)** | ***P-*value^a^** |
| --- | --- | --- | --- | --- | --- | --- |
| Clinical model A***** | 0.668(0.570-0.765) |  |  |  |  |  |
| +sCysC and uNAG | 0.784(0.703-0.865) | <0.001 | 0.104(0.042-0.166) | <0.001 | 0.660(0.335-0.985) | <0.001 |

*****Clinical model A for AKI prediction is composed of serum creatinine at ICU admission, need for vasopressor at ICU admission, APACHE II score; **^a^**Versus clinical model A.

**Abbreviations:** AKI, acute kidney injury; AUC-ROC, area under the receiver operating characteristic curve; NRI, net reclassification improvement index; IDI, integrated discrimination improvement index; CI, Confidence Interval; sCysC, serum Cystatin C; uNAG, urinary N-acetyl-ß-D-glucosaminidase; ICU, intensive care unit; APACHE II, Acute Physiology and Chronic Health Evaluation score.
