# Supplementary material for: A nomogram incorporating functional and tubular damage biomarkers to predict the risk of acute kidney injury for septic patients
Source: BMC Nephrol. 2021 May 13;22:176. doi: 10.1186/s12882-021-02388-w (PMC8120900; doi:10.1186/s12882-021-02388-w)
Supplement: Supplementary file 7 — (Table S6.) Correlations between AKI and mortality in entire cohort. [file 12882_2021_2388_MOESM7_ESM.docx]

**Supplementary Table 6 Correlations between AKI and mortality in entire cohort**

| **Spearman’s rho** | **ICU mortality (n)** | **In-hospital mortality (n)** | **30-day mortality (n)** |
| --- | --- | --- | --- |
| AKI (n) | 0.383**^*^** | 0.386**^*^** | 0.424**^*^** |
| Nomogram total score | 0.378**^*^** | 0.366**^*^** | 0.422**^*^** |

All correlations **^*^***P* <0.01.

Abbreviation: AKI, acute kidney injury; ICU, intensive care unit.
