# Supplementary material for: A nomogram incorporating functional and tubular damage biomarkers to predict the risk of acute kidney injury for septic patients
Source: BMC Nephrol. 2021 May 13;22:176. doi: 10.1186/s12882-021-02388-w (PMC8120900; doi:10.1186/s12882-021-02388-w)
Supplement: Supplementary file 8 — (Table S7.) Correlation between the total score calculated from the nomogram and thedayof AKIoccurrence after ICU admission. [file 12882_2021_2388_MOESM8_ESM.docx]

**Supplementary Table 7 Correlation between the total score calculated from the nomogram and the day of AKI occurrence after ICU admission**

| **Spearman’s rho** | **the day of AKI occurrence after ICU admission** |
| --- | --- |
| AKI patients |  |
| Total score | -0.264* |

Correlations **^*^***P* <0.05.

Abbreviation: AKI, acute kidney injury; ICU, intensive care unit.
