# Supplementary material for: A nomogram incorporating functional and tubular damage biomarkers to predict the risk of acute kidney injury for septic patients
Source: BMC Nephrol. 2021 May 13;22:176. doi: 10.1186/s12882-021-02388-w (PMC8120900; doi:10.1186/s12882-021-02388-w)
Supplement: Supplementary file 9 — (Table S8.) Predictive characteristics of SOFA and APCHE II for AKI prediction in the development cohort. [file 12882_2021_2388_MOESM9_ESM.docx]

**Supplementary Table 8 Predictive characteristics of SOFA and APACHE II for AKI prediction in the development cohort**

| **Logistic regression model** | **AUC-ROC^a^** | **Cut-off^b^** | **Se** | **Sp** | **+LR** | **-LR** | **PPV** | **NPV** |
| --- | --- | --- | --- | --- | --- | --- | --- | --- |
| **AKI (n = 69)** |  |  |  |  |  |  |  |  |
| **SOFA** | 0.680(0.603-0.756) | 6 | 0.52 | 0.79 | 2.43 | 0.61 | 0.51 | 0.80 |
| **APACHE II** | 0.701(0.631-0.772)^#^ | 19 | 0.70 | 0.64 | 1.92 | 0.48 | 0.45 | 0.83 |

**^a^**Values are presented as AUC-ROC (95% confidence interval); **^b^**Ideal cut-off value according to Youden’s index; ^#^*P*>0.05 vs. APACHE II.

**Abbreviations:** AKI, Acute kidney injury; AUC-ROC, area under the receiver operating characteristic curve; (+) LR, positive likelihood ratio; (-) LR, negative likelihood ratio; PPV, positive predictive value; NPV, negative predictive value; SOFA, sequential organ failure assessment score; APACHE II, Acute Physiology and Chronic Health Evaluation score.
