# Supplementary material for: A nomogram incorporating functional and tubular damage biomarkers to predict the risk of acute kidney injury for septic patients
Source: BMC Nephrol. 2021 May 13;22:176. doi: 10.1186/s12882-021-02388-w (PMC8120900; doi:10.1186/s12882-021-02388-w)
Supplement: Supplementary file 10 — (Table S9.) Logistic regression analysis of factors related to AKI in the development cohort including SOFA. [file 12882_2021_2388_MOESM10_ESM.docx]

**Supplementary Table 9 Logistic regression analysis of factors related to AKI in the development cohort including SOFA***

| Variable |  | Univariate analysis | |  |  | Multivariate analysis | |
| --- | --- | --- | --- | --- | --- | --- | --- |
|  | OR_unadj_ | 95% CI | *P* value |  | OR_adj_ | 95% CI | *P* value |
| Age, years | 1.006 | 0.989-1.025 | 0.476 |  |  |  |  |
| Males | 0.622 | 0.351-1.102 | 0.104 |  |  |  |  |
| BMI, kg/m2 | 0.985 | 0.901-1.077 | 0.735 |  |  |  |  |
| Preexisting clinical conditions |  |  |  |  |  |  |  |
| Hypertension | 1.248 | 0.690-2.256 | 0.464 |  |  |  |  |
| Diabetes mellitus | 1.349 | 0.626-2.907 | 0.444 |  |  |  |  |
| Cerebrovascular disease | 1.696 | 0.919-3.128 | 0.091 |  |  |  |  |
| Chronic liver disease | 2.044 | 0.602-6.937 | 0.251 |  |  |  |  |
| Coronary artery disease | 1.956 | 0.813-4.704 | 0.134 |  |  |  |  |
| Heart failure | 1.560 | 0.578-4.210 | 0.380 |  |  |  |  |
| Malignancy | 0.669 | 0.326-1.374 | 0.274 |  |  |  |  |
| CKD | 3.675 | 1.408-9.591 | 0.008 |  |  |  |  |
| COPD | 1.137 | 0.487-2.655 | 0.767 |  |  |  |  |
| Admission type, n (%) |  |  | 0.064 |  |  |  |  |
| Elective surgical (reference) |  |  |  |  |  |  |  |
| Emergency surgical | 3.080 | 0.990-9.578 | 0.052 |  |  |  |  |
| Medical | 3.300 | 1.214-8.970 | 0.019 |  |  |  |  |
| Medication before ICU admission, n (%) |  |  |  |  |  |  |  |
| Nephrotoxic drugs^a^ | 1.084 | 0.535-2.194 | 0.823 |  |  |  |  |
| Radiographic contrast | 2.073 | 0.818-5.255 | 0.125 |  |  |  |  |
| Sites of infection, n (%) |  |  | 0.982 |  |  |  |  |
| Pulmonary or thoracic cavity (reference) |  |  |  |  |  |  |  |
| Abdomen | 1.065 | 0.434-2.615 | 0.891 |  |  |  |  |
| Biliary tract | 1.597 | 0.259-9.864 | 0.614 |  |  |  |  |
| CNS infections | 0.856 | 0.292-2.508 | 0.776 |  |  |  |  |
| Others^b^ | 1.106 | 0.397-3.080 | 0.847 |  |  |  |  |
| MAP at ICU admission, mmHg | 1.002 | 0.985-1.019 | 0.833 |  |  |  |  |
| Need for vasopressor at ICU admission | 6.694 | 2.948-15.196 | <0.001 |  | 5.973 | 2.477-14.405 | <0.001 |
| Mechanical ventilation at ICU admission | 1.925 | 0.730-5.071 | 0.185 |  |  |  |  |
| Serum creatinine at ICU admission, mg/dL | 7.732 | 2.784-21.472 | <0.001 |  | 9.127 | 2.915-28.580 | <0.001 |
| Serum glucose at ICU admission, mg/dL | 1.004 | 0.999-1.008 | 0.093 |  |  |  |  |
| Hemoglobin at ICU admission, g/L | 0.996 | 0.985-1.007 | 0.454 |  |  |  |  |
| Platelet at ICU admission，10^9^/L | 0.997 | 0.994-1.000 | 0.070 |  |  |  |  |
| Serum PCT at ICU admission，ng/ml | 1.014 | 0.994-1.034 | 0.170 |  |  |  |  |
| CRP at ICU admission，mg/L | 1.002 | 0.998-1.006 | 0.287 |  |  |  |  |
| Total bilirubin at ICU admission >2mg/dL | 1.994 | 0.909-4.371 | 0.085 |  |  |  |  |
| Albumin at ICU admission <3 mg/dL | 1.160 | 0.660-2.038 | 0.606 |  |  |  |  |
| Table 2 Logistic regression analysis of factors related to AKI in the development cohort* (*Continued*) | | | | | | | |
| Variable |  | **Univariate analysis** | |  |  | **Multivariate analysis** | |
|  | **OR_unadj_** | **95% CI** | ***P* value** |  | **OR_adj_** | **95% CI** | ***P* value** |
| Lactate at ICU admission >2mmol/L | 0.996 | 0.531-1.871 | 0.991 |  |  |  |  |
| pH value at ICU admission ≤7.30 | 1.198 | 0.431-3.334 | 0.729 |  |  |  |  |
| SOFA score | 1.264 | 1.131-1.412 | <0.001 |  | 1.266 | 1.120-1.431 | <0.001 |
| UP, ml/kg/h | 1.142 | 0.893-1.460 | 0.289 |  |  |  |  |

^*^The clinical model was constructed without candidate variables of uNAG and sCysC in univariate logistic regression.

^a^includes any of the following medications administered within 5 days before ICU admission: nonsteroidal anti-inflammatory drug, angiotensin-converting enzyme inhibitor, angiotensin receptor blocker, immunosuppressant, sulfadiazine, aminoglycoside, vancomycin, acyclovir, amphotericin, allopurinol, or polymyxin; ^b^includes any of the following sites of infection: soft tissue, blood, or urinary tract.

**Abbreviations:** AKI, acute kidney injury; OR_unadj_, odds ratio unadjusted; OR_adj_, odds ratio adjusted; CI, confidence interval; BMI, body mass index; CKD, chronic kidney disease, defined as baseline eGFR<60 ml/min per 1.73m^2^; COPD, chronic obstructive pulmonary disease; CNS, central nervous system; MAP, mean arterial pressure; ICU, Intensive care unit; eGFR, estimated glomerular filtration rate; sCysC, serum Cystatin C; uNAG, urinary N-acetyl-ß-D-glucosaminidase; Cre, creatinine concentration; PCT, procalcitonin; CRP, C-reactive protein; SOFA, sequential organ failure assessment score; UP, urine production first 24 hours after admission; RRT, renal replacement therapy.
