# Supplementary material for: A nomogram incorporating functional and tubular damage biomarkers to predict the risk of acute kidney injury for septic patients
Source: BMC Nephrol. 2021 May 13;22:176. doi: 10.1186/s12882-021-02388-w (PMC8120900; doi:10.1186/s12882-021-02388-w)
Supplement: Supplementary file 11 — (Table S10.) AUC-ROC, NRI and IDI analyses of AKI in development cohort. [file 12882_2021_2388_MOESM11_ESM.docx]

**Supplementary Table 10 AUC-ROC, NRI and IDI analyses of AKI in development cohort**

| **Variables** | **AUC-ROC** | ***P-*value^a^** | **IDI (95% CI)** | ***P-*value^a^** | **cNRI (95% CI)** | ***P-*value^a^** |
| --- | --- | --- | --- | --- | --- | --- |
| Clinical model***** | 0.770(0.699-0.841) |  |  |  |  |  |
| +uNAG | 0.810(0.748-0.871) | 0.053 | 0.068(0.027-0.108) | <0.001 | 0.516(0.244-0.788) | <0.001 |
| +sCysC | 0.794(0.730-0.858) | 0.159 | 0.031(0.004-0.059) | 0.027 | 0.440(0.165-0.715) | 0.002 |
| +sCysC and uNAG | 0.830(0.773-0.886) | 0.014 | 0.086(0.043-0.129) | <0.001 | 0.694(0.438-0.961) | <0.001 |

*****Clinical model A for AKI prediction is composed of serum creatinine at ICU admission, need for vasopressor at ICU admission, SOFA score**; ^a^**Versus clinical model.

**Abbreviations:** AKI, acute kidney injury; AUC-ROC, area under the receiver operating characteristic curve; NRI, net reclassification improvement index; IDI, integrated discrimination improvement index; CI, Confidence Interval; sCysC, serum Cystatin C; uNAG, urinary N-acetyl-ß-D-glucosaminidase. ICU, intensive care unit.
