# Supplementary material for: A nomogram incorporating functional and tubular damage biomarkers to predict the risk of acute kidney injury for septic patients
Source: BMC Nephrol. 2021 May 13;22:176. doi: 10.1186/s12882-021-02388-w (PMC8120900; doi:10.1186/s12882-021-02388-w)
Supplement: Supplementary file 12 — (Table S11.) AUC-ROC of the AKI prediction model in the development cohort and the validation cohort. [file 12882_2021_2388_MOESM12_ESM.docx]

**Supplementary Table 11 AUC-ROC of the AKI prediction model* in the development cohort and the validation cohort**

| **Variables** | **AUC-ROC (95% CI)** | **95% CI** | ***P-*value** |
| --- | --- | --- | --- |
| **Development cohort** | 0.830±0.029 | 0.773-0.886 | <0.001 |
| **Validation cohort** | 0.776±0.042^a^ | 0.694-0.858 | <0.001 |

*****Prediction model for AKI prediction is composed of serum creatinine at ICU admission, need for vasopressor at ICU admission, SOFA score, sCysC, and uNAG**;** aAUC of the development cohort vs. AUC of t the validation cohort, Z = 1.058, *P* = 0.290.

**Abbreviations:** AKI, acute kidney injury; AUC-ROC, area under the receiver operating characteristic curve; CI, Confidence Interval; sCysC, serum Cystatin C; uNAG, urinary N-acetyl-ß-D-glucosaminidase. ICU, intensive care unit.
